# Predicting N Status in Maize with Clip Sensors: Choosing Sensor, Leaf Sampling Point, and Timing

**DOI:** 10.3390/s19183881

**Published:** 2019-09-09

**Authors:** Jose Luis Gabriel, Miguel Quemada, María Alonso-Ayuso, Jon I. Lizaso, Diana Martín-Lammerding

**Affiliations:** 1 Instituto Nacional de Investigación y Tecnología Agraria y Alimentaria (INIA-INAGEA), 28040 Madrid, Spain; 2 CEIGRAM, Universidad Politécnica de Madrid, 28040 Madrid, Spain

**Keywords:** chlorophyll, polyphenol, nutrient deficiency, fertilization, precision agriculture

## Abstract

Nitrogen (N) losses from agricultural systems increase air and water pollution, and these losses are highly correlated with the excessive fertilization. An adjusted N fertilization is then a key factor in increasing the N fertilizer efficiency, and leaf clip sensors can help to improve it. This study (combining five different field experiments in Central Spain) tried to identify the ability of the clip sensors in maize N status identification and yield prediction, comparing two different devices (SPAD-502^®^ and Dualex^®^) and identifying the best protocol for maize leaf sampling. As a result, the study demonstrated that different leaf clip chlorophyll sensors presented similar results, although some differences appeared at larger N concentrations. Complementary polyphenol information (as flavonol) can improve the maize N deficiency prediction. Moreover, valuable information for a proper sampling protocol was obtained with this study. It proved that the sampling position (in the leaf and in the plant) and sampling time were crucial for a better estimation of the maize N status. Proper fertilization recommendations could be achieved based on clip chlorophyll sensor measurements.

## 1. Introduction

Nitrogen (N) losses from agricultural systems are a major problem for air (NH_3_ and N oxides [1]) and water (NO_3_ [2]) pollution. These losses are highly correlated to the excessive fertilization [3]. Therefore, adjusting the N fertilization rate to the crop demand and N actual status is a key factor for improving the N fertilizer efficiency of the system [4]. The N status can be measured based on a soil or plant analysis, but those are destructive methods that demand high economic, human and time resources. As an alternative, there are rapid and non-destructive methods based on optical properties, which can provide a reliable estimation of the crop N status [5,6]. Based on this N status, N fertilization side dressings can be adapted for each year or for the different areas in the farm. In a maize crop, this corrective side dressing can be applied until the maize presents around 8 leaves (machinery limitation) or even later if fertigation is available.

Substantial research on technologies for N status prediction has been accomplished on these optical methods during recent years. There is information for many crops, such as cereals (maize (*Zea maize* L.) [5,7,8] or wheat (*Triticum aestivum* L.) [4,9]), vegetables (tomato (*Solanum lycopersicum* L.) [8] or cucumber (*Cucumis sativus* L.) [10]), sunflower (*Helianthus annuus* L.) [11], potato (*Solanum tuberosum* L.) [12] or pastures (such as festulolium (*Festulolium braunii* K. Richert A. Camus) [13]). Fox and Walthall [14] and Hunt et al. [15] showed that the greenness of the leaves is highly correlated with the chlorophyll content, and also with their N content. Chlorophyll content is key in the photosynthesis process [13]. The leaf clip equipment to estimate the chlorophyll content relies mainly on the difference between the leaf transmittance at two wavelengths. Moreover, the clip system ensures a good contact with the plant tissue and an independence to external light conditions, but on the other hand the sampled surface is small [16]. These sensors are already at a commercial level thanks to their recognized potential for fast N fertilizer recommendations [17,18]. However, this technique presents some limitations: (i) a non-uniform leaf and plant chlorophyll distribution [19], (ii) the crop development status when readings are taken [20], and (iii) nutrient deficiencies other than N [14]. 

Related to the interferences among various nutrient deficiencies or stress conditions, some devices also provide optical measurements of concentrations of polyphenols or other substances in the leaf [21,22]. These secondary metabolites are compounds that usually tend to increase in leaves under stress conditions and can help to improve the estimation of the N status estimation [23,24]. The timing of the measurement and position on the leaf have received attention. Some authors have observed that crops such as potato try to maintain the chlorophyll concentration per leaf surface unit, so N deficiencies produce smaller leaves, which, however, are not less green [12]. However, other crops such as maize develop an opposite strategy, keeping the leaf surface almost constant (differences smaller than 30%), but with large differences in the chlorophyll concentration per surface unit [25]. It is under this latest condition that the optical sensors are more reliable. Moreover, Lemaire and Gastal [26] observed that there was a decrease in the plant N concentration as the aboveground biomass increased and that there was a critical level below which the yield could be compromised. Consequently, the plant size and crop phenology must be considered when programing N fertilization.

Maize crop has been ranked by the FAO (United Nations Food and Agriculture Organization) as the first cereal in terms of production, and as the second in terms of surface [27]. The maize world production is constantly increasing, reaching 1135 million Mg in 2017, grown on almost 200 million ha. Nitrogen content in this crop represents only around 2.4 % of the total dry mass. However, the availability of water and N are considered the most limiting factors for the grain yield [28]. Moreover, since it is a crop that does not cover the soil completely at earlier stages, when proper fertilization is crucial, soil noise interferes strongly with remote sensing recommendations [5]. Therefore, clip sensors could be an interesting option for adjusting crop fertilization and reducing worldwide agricultural pollution problems quite efficiently.

The main goal of these experiments was to determine the ability of the clip sensors to identify the maize N status. For that purpose, we focused on some specific objectives, including (i) comparing the performance of various devices, (ii) identifying the best protocol for maize leaf sampling, and (iii) establishing the accuracy of the N status prediction and the chlorophyll indexes as yield estimators.

## 2. Materials and Methods

This study includes the results obtained during five different years, since 2006, in five maize field trials located in the Madrid Region (Central Spain). These trials compared various N managements providing a diversity of maize N statuses, valuable for the methodological comparisons proposed in this work. In addition, other management variables were also different (tillage, cover crops, irrigation...). The chlorophyll sensors that were used were SPAD-502^®^ (Konica Minolta Inc., Japan) and/or Dualex^®^ Scientific (Force-A, Orsay, France). Both are leaf clip sensors that estimate the chlorophyll content based on the difference of LED light transmitted through a leaf at red (~650 nm) and infrared (~940 nm) wavelengths within a small dark chamber (~1 cm^2^) [29]. The dimensionless index obtained with both sensors will be called SPAD (for SPAD-502^®^) and Chl (for Dualex^®^) throughout the manuscript. In addition, the Dualex^®^ Scientific also measures the leaf polyphenol concentration as flavonol (Flav) and the Nitrogen Balance Index (NBI). This Flav is directly related to the optical absorption of the leaf epidermis under UV light. In this case, the red light was compared with the UV wavelength (~370 nm) transmitted through the leaf epidermis. The NBI was calculated as the ratio between the Chl and Flav content, providing an estimation of the N nutritional status [22,24]. As the main goal was to predict the maize N status, the actual chlorophyll content was not measured in the leaves.

### 2.1. Comparison between Sensors

In order to compare the results obtained with both sensors, the data from three experiments (Experiments 1, 2 and 3; Table 1) were merged. Simultaneous measurements of SPAD and Chl were obtained from the last fully expanded leaf on 15 representative plants in the central zone of each plot. Experiment 1 consisted of six N fertilizer rates from 0 to 200 kg N ha^−1^ (as ammonium nitrate) at 40 kg N ha^−1^ intervals, with four replications, resulting in 24 plots. Further information about this experimental design or the crop results can be found in Quemada et al. [7]. Experiment 2 consisted of the same six N fertilizer rates as the previous one, but with six replications, resulting in 36 plots. Experiment 3 consisted of five N fertilizer rates (0, 70, 120, 170 and 220 kg N ha^−1^ as calcium ammonium nitrate) and two irrigation rates with six replications, resulting in 60 plots. Further information about this experimental design or the crop results can be found in Gabriel et al. [5]. All plots were measured on two dates, corresponding with the second N dressing (middle of June and around 6 fully expanded leaves) and anthesis (end of July). In addition, in the first experiment at anthesis, measurements from the ear leaf were also conducted. The results for each of the 15 sampling points per plot, for the average plot values, and for the relative standard errors of each sensor were analysed in this study.

### 2.2. Leaf Sampling Selection

The relevance of selecting the sampling position to obtain an accurate measurement was tested by comparing readings from both: the sampling position on the leaf and the sampled leaf. A detailed study with Dualex^®^ Scientific (Experiment 4; Table 1) was developed [30], to determine the adequate sampling position on the leaf. Drip irrigated maize was sown on April 17th at 75,000 plant ha^−1^. The experiment had two treatments, with or without fertigation (as calcium ammonium nitrate), and three replications. On each plot, two representative plants were evaluated through the entire cycle. In order to determine the best sampling position along the maize leaf, measurements were done at flowering (July 10th) on the ear leaf from the base to the tip at 1-cm intervals. On average, fertigated ear leaves were 77 cm long and not fertigated ear leaves were 71 cm long. For comparison purposes, measurements were made relative to the total leaf length. 

In order to choose the best leaf to measure, all green leaves were evaluated for the 12 selected plants at 5 dates (June 8th, July 3rd, July 10th, August 5th and August 21st). Each measurement consisted of two samples per leaf, both done in the middle of the leaf but separated by around 1 cm each. For the result interpretation, three analyses were done: (1) the variability across leaves on the same date, (2) the evaluation of the relative standard error across leaves, and (3) the evolution of the nutrition indexes in the same leaves throughout the time, selecting in this case the leaves 9, 14 (ear) and 20 (flag). For this experiment, the Flav and the NBI were analysed in addition to the Chl value in order to improve the recommendation of the leaf to be sampled.

### 2.3. Greenness as Yield Predictor

In order to evaluate the chlorophyll indexes as yield predictors, data from three experiments (Experiments 1, 2 and 5; Table 1) were merged. In these experiments, SPAD measurements were collected at four different dates, corresponding to the first and second N dressing (middle of May and June, respectively), flowering (end of July) and middle of August. Experiment 5 consisted of five precedent winter treatments before summer maize (vetch, barley, rapeseed, fallow and a control without cover crop nor fertilization) with four replications, resulting in 20 plots per year. Further information about the experimental design or the crop results can be found in Gabriel and Quemada [31]. Experiments 1 and 2 were already defined in the *Comparison Between Sensors* section. Each experiment was sampled with SPAD fifteen times per plot on the last fully expanded leaf before flowering, on the ear and flag leaves at flowering, and on the ear leaf during the grain filling in August. The SPAD measurements were compared with the plot final yield at harvest. The yield was obtained with an experimental combine harvester in two 10 m-rows, from each plot, and the grain was oven-dried (65 °C) for the dry matter weight. Combining all experiments and plots, the comparison resulted in 120 points per date and leaf. However, there were data for August only from Experiment 1 in 2007 and 2008, and from Experiment 3 (resulting in 76 points). Additionally, there were data for the ear leaf at flowering only from Experiment 2 (resulting in 24 points), and there were data from Experiments 1 and 3 for May (resulting in 96 points). The results for each sampling date and leaf were fitted to second order polynomial curves.

### 2.4. Greenness as Crop N Status Predictor

We conducted two comparisons to evaluate the chlorophyll indexes as N status predictors. The first one compared the SPAD and Chl measurements with the N concentration measured in the same leaf, using our data from Experiment 3 (Table 1). Further information about the N concentration measurement can be obtained in Gabriel et al. [5]. The second comparison was SPAD with the final N uptake by maize at harvest. In this case, we used data from Experiments 1 and 5. The N uptake was calculated by multiplying the grain weight and the rest of the aboveground biomass by their respective N concentrations. Further information on these data can be found in Gabriel and Quemada [31] and in Quemada et al. [7].

### 2.5. Statistical Analysis

To quantify the degree of correlation between data from sensors and agronomic measurements, different models (from linear to polynomic, exponential and logarithmic) were fitted, and the coefficient of determination (R^2^) was calculated to analyse the goodness of fit. Tukey post-hoc tests were used to separate the means between treatments (P ≤ 0.05). Statistical analyses were conducted with R.3.5.1 [32].

## 3. Results and Discussion

### 3.1. Comparison between Sensors

**Figure 1:**
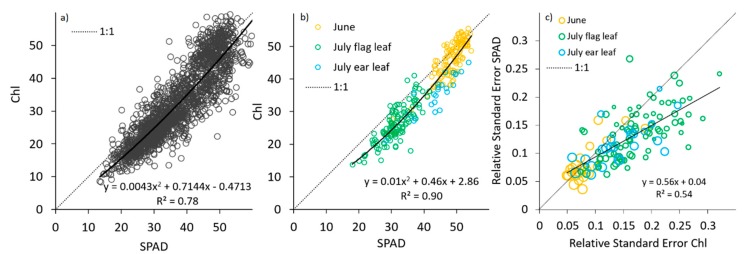
The relationship between the average measurements (from 15 samples) obtained with SPAD and Dualex (Chl) in June on the last completely expanded leaf (N = 120), in July on the flag leaf (N = 120) and in July on the ear leaf (N = 24) from Experiments 1, 2 and 3. (**a**) Including all individual measurements (N = 3960); (**b**) Aggregating by plot, date and leaf (N = 264); (**c**) Comparing the relative standard error observed in the plot (larger circles represent larger original average values; N = 264).

The relationship between the average SPAD measurements and Chl was strong (R^2^ = 0.90; Figure 1), even when various experiments, growth stages or leaves were included in the comparison, similar to the relationship obtained by Tremblay et al. [33]. As observed by Casa et al. [34], the correlation was not linear. When the measured values were low, the SPAD tended to provide higher readings than the Chl (SPAD ≈ 5 points higher than Chl). In addition, SPAD tended to saturate earlier than Dualex when the chlorophyll content increased; therefore, the SPAD were lower than the Chl readings at high values. When single readings from the same leaf were analysed, the correlation was reduced to 0.78 (Figure 1), mostly because of the internal leaf variability. This result strengthens the idea that average readings from a representative number of leaves provide more accurate results than the single ones. At low values, SPAD presented a higher precision (with a lower relative standard error) than Dualex, whereas both sensors presented a similar precision at high values (Figure 1). Although, in general, the NBI Dualex index can improve the N status prediction with respect to Chl [5,22], a comparison of NBI with SPAD at different dates or sampled leaves did not provide a good correlation (R^2^ = 0.36).

### 3.2. Leaf Sampling Selection

**Figure 2:**
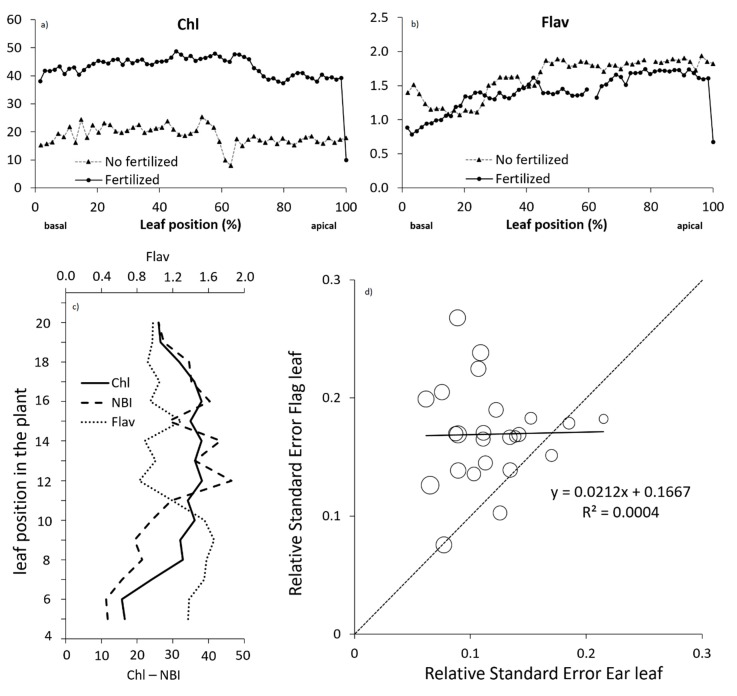
Measurements obtained with Dualex (dimensionless) at different positions during maize anthesis. (**a**) Chl and (**b**) Flav measured along the ear leaf in fertilized or not fertilized maize plants, (**c**) Chl, Flav and NBI measured on each leaf (from the lower remaining leaf to the flag leaf; ear leaf: 14) in a fertilized maize and (**d**) Chl relative standard error observed at the flag leaf and at the ear leaf (larger circles represent larger original Chl values).

Regarding the position along the leaf, the largest differences in the Chl were observed in the apical 1/3, closer to the tip, where the values decreased (Figure 2a). However, the Flav was lower in the basal 1/3, closer to the leaf collar (Figure 2b). Because of that, the mid-section of the leaf was the best sampling position in order to achieve the larger differences between both N treatments.

With respect to the leaf that should be measured in the plant (measured at anthesis because all the leaves were completely expanded), the largest Chl value was obtained in the leaves located closer to the ear leaf (Figure 2c). It is interesting to notice that sometimes there were some saw peaks between even- and odd-numbered leaves related to the plant orientation with respect to the sun. In some plants, the peak was in the even leaves, while in others it was in the odd ones. In any case, the Chl values obtained from the leaf number 8 to the leaf number 18 were, on average, relatively stable. The Flav presented a different pattern, with larger values in the leaves of the lower half of the plant. In this case, there was also a saw peaks distribution, but the Flav peaks corresponded to the Chl valleys. The relative standard error between samples (obtained from the ear leaf and from the flag leaf at flowering and in the same plants) was higher in the measurements obtained in the flag leaves (Figure 2d). Considering all this information, and as occurred in the position within the leaf, the intermediate leaves presented the most representative value. Based on that, the ear leaf would be an interesting leaf to measure in order to increase the replicability between different plants. In any case, several plants should be measured to avoid the bias produced by the saw peaks.

**Figure 3:**
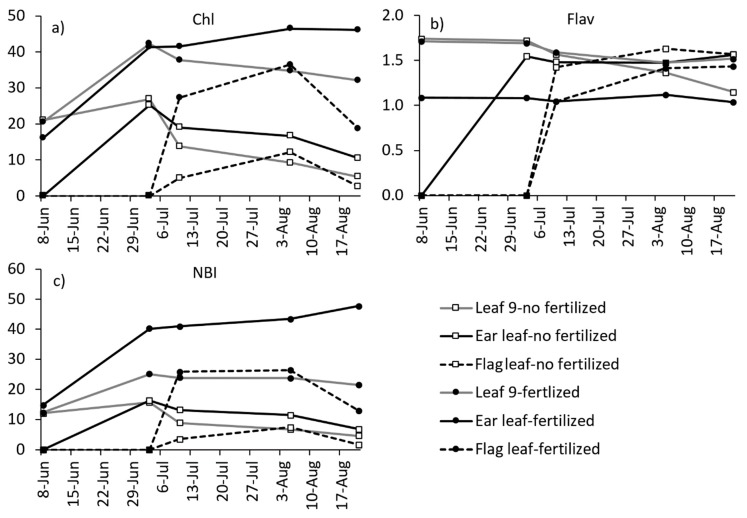
The average measurements (N = 6) obtained with Dualex (dimensionless) at different leaves (9, 14-ear and 20-flag) along the maize growing season for: (**a**) the chlorophyll index (Chl), (**b**) the flavonol index (Flav) and (**c**) the N balance index (NBI) at two N rates.

Finally, the leaf age when the leaf was measured affected the reading if the leaf was not completely expanded or if it had started senescence (Figure 3). However, the study conducted comparing the leaves 9, 14 (ear leaf) and 19 (usually the previous one to the flag leaf) in various plants and dates showed that once the leaf reached a maximum size (complete expansion), variations in the Chl or Flav content were small until senescence (Figure 3). Moreover, differences in the Chl content between treatments across leaves and dates were uniform (Figure 3a). However, differences in the Flav content between treatments were larger in the ear leaf and increased with time, making it easier to detect fertilizer deficiencies measuring this leaf (Figure 3b). The NBI combined the Chl and Flav results, thus maximizing the N treatment differences, and even increasing the differences in time (Figure 3c). Padilla et al. [10] and Tremblay et al. [33] also observed that the NBI could provide more robust results for the N status prediction than Chl or Flav independently.

### 3.3. Greenness as Yield Predictor

**Figure 4:**
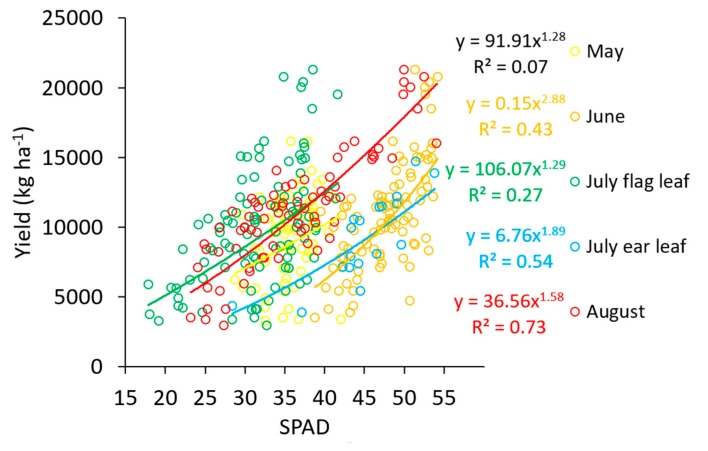
A comparison between the SPAD readings (dimensionless) at four different dates (May, June, July or August) and the final yield (as grain dry matter). Each circle is the average of 15 SPAD readings per plot and was obtained from the last fully expanded leaf in May (N = 96) and June (N = 120), from the flag (N = 120) or from the ear leaf (N = 24) in July, and from the ear leaf in August (N = 76).

The chlorophyll content estimations with sensors could be interesting for the yield estimation [34]. The experiment where the chlorophyll content was estimated with SPAD at four dates provided information about the accuracy obtained with this sensor (Figure 4). The best fit was obtained with exponential curves. Although the curves were almost linear along the observed range, the exponential fitted better because some saturation occurred at high SPAD values. A similar saturation effect was observed by Quemada et al. [7] when maize growth was predicted with Chl and SPAD. At the first dressing date (May), the correlation between SPAD and the final yield was very low (R^2^ = 0.10). This was because the observed SPAD range was very narrow (between 30 and 40 for most of the plots) and because the maize still had time to correct the deficiencies. At the second dressing date (June), the correlation increased to R^2^ = 0.45, still suggesting a capability to recover potential yields with further fertilization. At flowering (July), the prediction of the final yield could rely on readings from either the flag or from the ear leaf. The correlation between the yield and the flag leaf readings was very poor (R^2^ = 0.29), mostly because of the size variability between the flag leaves and because the leaf senescence started soon after silking. However, the correlation between the yield and ear leaf SPAD increased to R^2^ = 0.54. Finally, the SPAD measurements done during August increased the yield prediction to R^2^ = 0.73. Therefore, the yield prediction with chlorophyll sensors is possible, although the accuracy increases when the measurement is done closer to harvest. These results could support the correction of N deficiencies at late growth stages in cropping systems that allow a late N application (i.e., by fertigation), as Padilla et al. [10] suggested. However, they could also help to get better estimations of the potential yield before harvest, improving harvest machinery scheduling or an accurate appraisal by insurance companies. 

### 3.4. Greenness as N Status Predictor

**Figure 5:**
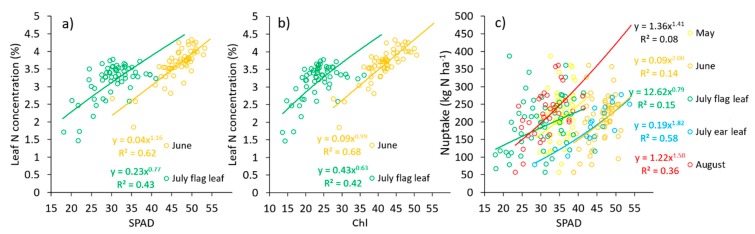
A comparison between the (**a**) SPAD and (**b**) Dualex readings (dimensionless) with the N concentration in the same leaf at two different dates, and between (**c**) the SPAD readings and the final N uptake by the plant at four dates (May, June, July or August). Each circle is the average of 15 SPAD or Dualex readings per plot and was obtained from the last fully expanded leaf.

The chlorophyll content has previously been pointed out as an N status predictor [35,36]. In this study, the chlorophyll content as an N status predictor presented some potentialities but also some deficiencies (Figure 5). On the one hand, both sensors predicted the N concentration in the leaves with a similar accuracy (R^2^ = 0.62 and 0.68 for SPAD and Chl, respectively, in June, and R^2^ = 0.43 and 0.42 in July; Figure 5a,b). However, the sensors estimation was not comparable across dates or leaves. Thus, the further development of the measurement protocol should be incorporated in the future to improve comparability across experiments. With respect to the N uptake prediction (Figure 5c), the results were similar to the results obtained for the yield prediction (Figure 4). However, some important reductions in the relationship in the August and June predictions were found. The highest accuracy was found when the earl leaf was sampled at flowering, with R^2^ = 0.58.

## 4. Conclusions

The clip chlorophyll sensors have shown the ability to adequately identify the maize N status at various crop developmental stages. Moreover, different clip chlorophyll sensors, as SPAD and Dualex, exhibited comparable results, even when evaluating small differences. However, complementary information such as flavonols can improve maize N status information. Relative to the best sampling protocol, it is interesting to know that once the leaf was completely expanded, the differences in the chlorophyll or flavonol content did not change until senescence. However, there were differences across the leaves, depending on their position along the plant and on their orientation. Differences were also detected within the same leaf. Because of that, the best recommendation is to always sample in the ear leaf (or in the last leaf that has completely expanded before ear leaf expansion), in the mid-section on the leaf, and in a representative number of leaves. Finally, clip chlorophyll sensors were able to predict the yield and N status, improving the prediction accuracy as the crop development progressed. However, a single correlation equation was not found, and results were highly dependent on the crop stage and on the sampled leaf, so further studies should be done in order to standardize a general sampling protocol. In any case, clip chlorophyll sensors provide valuable information on the variation of the N content inside a field, when all the maize plants are at the same growth stage.

## Figures and Tables

**Table 1:** Summary of the five experiments used for the three analyses.

Code	Location	Years	Treatments	Sensor	Sampling Dates	Agronomic Results Reference
1	Aranjuez (40°03′N, 03°31′W)	2012	6 N rates by 4 rep.	SPAD Dualex	June (2nd dr.) July (fl.)	Quemada et al., 2014 [7]
2	Madrid (40°27′N, 03°44′W)	2012	6 N rates by 6 rep.	SPAD Dualex	June (2nd dr.) July (fl.)	
3	Aranjuez (40°03′N, 03°31′W)	2015	5 N rates by 2 irrigation by 6 rep.	SPAD Dualex	June (2nd dr.) July (fl.)	Gabriel et al., 2017 [5]
4	Alcalá de Henares (40°32′N, 3°20′W)	2015	2 N rates by 3 rep.	Dualex	June July (by 2; fl.) August (by 2)	Guardia et al., 2017 [30]
5	Aranjuez (40°03′N, 03°31′W)	2007, 2008, 2009	5 cover crops by 4 rep.	SPAD	May (1st dr.) June (2nd dr.) July (fl.) August	Gabriel & Quemada, 2011 [31]

* Abbreviations: rep. (replications), dr. (dressing), fl. (flowering).

## References

[1] Snyder C.S., Bruulsema T.W., Jensen T.L., Fixen P.E. (2009). Review of greenhouse gas emissions from crop production systems and fertilizer management effects. Agric. Ecosyst. Environ..

[2] Quemada M., Baranski M., de Lange M.N.J., Vallejo A., Cooper J.M. (2013). Meta-analysis of strategies to control nitrate leaching in irrigated agricultural systems and their effects on crop yield. Agric. Ecosyst. Environ..

[3] Quemada M., Gabriel J.L. (2016). Approaches for increasing nitrogen and water use efficiency simultaneously. Glob. Food Secur..

[4] Arregui L.M., Quemada M. (2008). Strategies to improve nitrogen-use efficiency in winter cereal crops under rainfed Mediterranean conditions. Agron. J..

[5] Gabriel J.L., Zarco-Tejada P.J., López-Herrera P.J., Pérez-Martín E., Alonso-Ayuso M., Quemada M. (2017). Airborne and ground level sensors for monitoring nitrogen status in a maize crop. Biosyst. Eng..

[6] Padilla F.M., Gallardo M., Peña-Fleitas M.T., De Souza R., Thompson R.B. (2018). Proximal optical sensors for nitrogen management of vegetable crops: A review. Sensors.

[7] Quemada M., Gabriel J.L., Zarco-Tejada P. (2014). Airborne hyperspectral images and ground-level optical sensors as assessment tools for maize nitrogen fertilization. Remote Sens..

[8] Kalaji M.H., Dąbrowski P., Cetner M.D., Samborska L.A., Łukasik I., Brestic M., Zivcak M., Horaczek T., Mojski I., Kociel H. (2017). A comparison between different chlorophyll content meters under nutrients deficiency conditions. J. Plant Nutr..

[9] Ravier C., Quemada M., Jeuffroy M.H. (2017). Use of a chlorophyll meter to assess nitrogen nutrition index during the growth cycle in winter wheat. Field Crop. Res..

[10] Padilla F.M., Peña-Fleitas M.T., Gallardo M., Thompson R.B. (2016). Proximal optical sensing of cucumber crop N status using chlorophyll fluorescence indices. Eur. J. Agron..

[11] Alonso-Ayuso M., Gabriel J.L, Quemada M. (2016). Nitrogen use efficiency and residual effect of fertilizers with nitrification inhibitors. Eur. J. Agron..

[12] Vos J., van der Putten P.E.L. (1998). Effect of nitrogen supply on leaf growth, leaf nitrogen economy and photosynthetic capacity in potato. Field Crops Res..

[13] Mastalerczuk G., Borawska-Jarmułowicz B., Kalaji H.M, Dąbrowski P., Paderewski I. (2017). Gas-exchange parameters and morphological features of festulolium (Festulolium braunii K. Richert A. Camus) in response to nitrogen dosage. Photosynthetica.

[14] Fox R.H., Walthall C.L., Schepers J.S., Raun W.R. (2008). Crop Monitoring Technologies to Assess Nitrogen Status. Nitrogen in Agricultural Systems, Agronomy Monograph 49.

[15] Hunt E.R., Doraiswamy P.C., McMurtrey J.E., Daughtry C.S.T., Perry E.M., Akhmedov B. (2013). A visible band index for remote sensing leaf chlorophyll content at the canopy scale. Int. J. Appl. Earth Obs..

[16] Arregui L.M., Lasa B., Lafarga A., Irañeta I., Baroja E., Quemada M. (2006). Evaluation of chlorophyll meters as tools for N fertilization in winter wheat under humid Mediterranean conditions. Eur. J. Agron..

[17] Samborski S.M., Tremblay N., Fallon E. (2009). Strategies to make use of plant sensors-based diagnostic information for nitrogen recommendations. Agron. J..

[18] Piekielek W.P., Fox R.H., Toth J.D., Macneal K.E. (1995). Use of a chlorophyll meter at the early dent stage of corn to evaluate nitrogen sufficiency. Agron. J..

[19] Monje O.A., Bugbee B. (1992). Inherent limitations of nondestructive chlorophyll meters: A comparison of two types of meters. HortScience.

[20] Martínez D.E., Guimat J.J. (2004). Distortion of the SPAD 502 chlorophyll meter readings by changes in irradiance and leaf water status. Agronomie.

[21] Cerovic Z.G., Ounis A., Cartelat A., Latouche G., Goulas Y., Meyer S., Moya I. (2002). The use of chlorophyll fluorescence excitation spectra for the non-destructive in situ assessment of UV absorbing compounds in leaves. Plant Cell Environ..

[22] Tremblay N., Wang Z., Cerovic Z.G. (2011). Sensing crop nitrogen status with fluorescence indicators: A review. Agron. Sustain. Dev..

[23] Hamilton J., Zangerl A., De Lucia E., Berenbaum M. (2001). The carbon-nutrient balance hypothesis: Its rise and fall. Ecol. Lett..

[24] Cartelat A., Cerovic Z.G., Goulas Y., Meyer S., Lelarge C., Prioul J.L., Barbottin A., Jeuffroy M.H., Gate P., Agati G. (2005). Optically assessed contents of leaf polyphenolics and chlorophyll as indicators of nitrogen deficiency in wheat (Triticum. aestivum L.). Field Crop. Res..

[25] Vos J., van der Putten P.E.L., Birch C.J. (2005). Effect of nitrogen supply on leaf appearance, leaf growth, leaf nitrogen economy and photosynthetic capacity in maize (Zea mays L.). Field Crop. Res..

[26] Lemaire G., Gastal F., Lemaire G. (1997). N uptake and distribution in plant canopies. Diagnosis of the Nitrogen Status of Crops.

[27] FAO (2019). Food and Agriculture Organization of the United Nations; Statistic Division. http://faostat.fao.org/.

[28] Elazab A., Ordóñez R.A., Savin R., Slafer G.A., Araus J.L. (2016). Detecting interactive effects of N fertilization and heat stress on maize productivity by remote sensing techniques. Eur. J. Agron..

[29] Yadava U.L. (1986). A rapid and nondestructive method to determine chlorophyll in intact leaves. Hortscience.

[30] Guardia G., Cangani M.T., Andreu G., Sanz-Cobena A., García-Marco S., Álvarez J.M., Recio-Huetos J., Vallejo A. (2017). Effect of inhibitors and fertigation strategies on GHG emissions, NO fluxes and yield in irrigated maize. Field Crop. Res..

[31] Gabriel J.L., Quemada M. (2011). Replacing bare fallow with cover crops in a maize cropping system: Yield, N uptake and fertiliser fate. Eur. J. Agron..

[32] R Core Team (2018). R: A Language and Environment for Statistical Computing. R Foundation for Statistical Computing. http://www.R-project.org/.

[33] Tremblay N., Wang Z., Bélec C. (2007). Evaluation of the Dualex for the assessment of corn nitrogen status. J. Plant Nutr..

[34] Casa R., Castaldi F., Pascucci S., Pignatti S. (2015). Chlorophyll estimation in field crops: An assessment of handheld leaf meters and spectral reflectance measurements. J. Agr. Sci..

[35] Wood C.W., Reeves D.W., Himelrick D.G. (1993). Relationships between chlorophyll meter readings and leaf chlorophyll concentration, N status, and crop yield—A review. Proc. Agron. Soc. New Zealand.

[36] Daughtry C.S.T., Walthall C.L., Kim M.S., de Colstoun E.B., McMurtrey J.E. (2000). Estimating corn leaf chlorophyll concentration from leaf and canopy reflectance. Remote Sens. Environ..

